# Study on the Preparation and Conjugation Mechanism of the Phosvitin-Gallic Acid Complex with an Antioxidant and Emulsifying Capability

**DOI:** 10.3390/polym11091464

**Published:** 2019-09-07

**Authors:** Bin Jiang, Xiaojing Wang, Linlin Wang, Shuang Wu, Dongmei Li, Chunhong Liu, Zhibiao Feng

**Affiliations:** 1 Department of Applied Chemistry, Northeast Agricultural University, NO.600 Changjiang Road Xiangfang District, Harbin 150030, China (B.J.) (X.W.) (L.W.) (D.L.) (C.L); 2 Heilongjiang Eco-meteorology Center, NO.71 Diantan Road Xiangfang District, Harbin 150030, China

**Keywords:** phosvitin, gallic acid, conjugation, antioxidant activity, emulsifier, mechanism

## Abstract

To develop a novel emulsifier with an antioxidant capacity, a phosvitin-gallic acid (Pv–GA) complex was prepared via a free-radical method. This emulsifier characterizes some key technologies. Changes in the molecular weight of the Pv–GA complex were analyzed by sodium dodecyl sulfate polyacrylamide gel electrophoresis (SDS–PAGE) and the matrix-assisted laser desorption/ionization time of light mass spectrometry (MALDI-TOF-MS). Fourier transform infrared spectroscopy (FTIR) indicated that C=O, C–N and N–H groups were also likely to be involved in the formation of the complex. A redshift was obtained in the fluorescence spectrogram, thereby proving that the covalent combination of Pv and GA was a free radical-forming complex. The results indicated that Pv and GA were successfully conjugated. Meanwhile, the secondary structure of Pv showed significant changes after conjugation with GA. The antioxidant activity and emulsifying properties of the Pv–GA complex were studied. The antioxidant activity of the Pv–GA complex proved to be much higher than that of the Pv, via assays of the scavenging activities of 2,2-Diphenyl-1-picrylhydrazyl (DPPH) and 2,2’-azino-bis(3-ethylbenzothiazoline-6-sulfonic acid) (ABTS) radicals and because of their ability to reduce power. The emulsification activity of the Pv–GA complex was also slightly higher than that of Pv. To function with the most demanding antioxidant and emulsification activities, the optimum conjugation condition was Pv (5 mg/mL) conjugated 1.5 mg/mL GA. Furthermore, the mechanism of Pv–GA conjugation was studied. This study indicated that GA could quench the inner fluorescence of Pv, and this quenching was static. There was a strong interaction between GA and Pv, which was not obviously affected by the temperature. Furthermore, several binding sites were close to 1, indicating that there was an independent class of binding sites on Pv for GA at different temperatures. The conjugation reaction was a spontaneous reaction, and the interaction forces of GA and Pv were hydrogen bonds and van der Waals force.

## 1. Introduction

Egg yolk proteins are widely applied in the food industry due to their remarkable emulsifying properties [1]. About 11% of the total yolk protein is phosvitin (Pv) [2]. Pv is a phosphoprotein containing about 10% phosphorus [3]. The molecular weight of Pv is about 35 kDa. Moreover, Pv exhibits a high degree of hydrophilicity, which is related to its structural characteristics. Pv contains a large amount of phosphoserine clusters, and the C-terminal of only 15 residues is rich in hydrophobic groups [4]. Thus, Pv exhibits a satisfactory emulsifying capacity and could act as a good emulsifier (both with a high emulsifying activity and emulsion stability) [2]. However, Pv is easily oxidized, resulting in loss of emulsifying ability. Thus, native Pv is insufficient to meet the standards of both emulsification and inhibiting oxidation (i.e., being an antioxidant). Protecting Pv from oxidation was necessary to broaden its application in emulsions. Conjugation with antioxidant compounds could significantly increase the radical scavenging activities of Pv without loss of its emulsifying activities and emulsion stability [5]. Pv utilized in combination with resveratrol improves antioxidant potential in emulsion systems [6]. Nakamura and others [7] reported that the conjugation of Pv and galactomannan enhanced the antioxidant activity of Pv. 

Polyphenols are widely distributed in plants; they are composed of a large number of secondary metabolites and rich in nature [8]. Polyphenols contain aromatic rings with one or more hydroxy substituents, ranging from simple polyphenols to highly polymerized compounds [9]. They are widely accepted as natural antioxidants and related to a lower incidence of diseases that are considered to be caused by free radicals [10]. Polyphenols are known as being essential substances for human health that could reduce the damage caused by free radicals. Gallic acid (GA) is proven to be a phenolic acid with antioxidant ability [11]. It can be found in many plants and is usually used in meat production [12]. GA is widely used as an important natural antioxidant in the food and pharmaceutical industries [13]. Previous studies have demonstrated that the conjugation of GA and proteins could remarkably improve the antioxidant properties of proteins. 

There are two modes of interactions between phenolic compounds and proteins: non-covalent linkage and covalent linkage [8]. In the free radical method, phenolic compounds and proteins conjugated via non-covalent linkage and hydrogen peroxide ascorbic acid acted as an initiator. This method was proven to be a fast, simple, and effective polyphenol-protein binding method [14] and could avoid the degradation of polyphenols [15]. Moreover, metallic iron or an organic solvent was not required in this method, and the complex could be easily collected and purified [16]. Many studies on proteins and polyphenols conjugated by free radical methods were reported. Liu studied the lactoferrin-polyphenol conjugates generated by free-radical graft copolymerization [8]. GA-chitosan and catechin-chitosan conjugates were prepared by free radical methods [17]. However, no report has focused on the interaction between the Pv and GA conjugated by a free radical method.

The conjugation of two substances would change the structure and characteristics of the substance itself. Studies on this conjugation mechanism are essential for understanding the cause of these changes. Some studies have described the interactions between ligands and bio-macromolecules. The process of the binding of carbamazepine and bovine serum albumin is a spontaneous molecular interaction and is mainly driven by hydrophobic force [18]. Structural analysis indicated that both the interactions, hydrophilic and hydrophobic, play important role in the in the process of polyphenols bound with *β*-lactoglobulin through two approaches (means) [19]. The conjugation of polyphenols and proteins could improve the antioxidant properties of proteins. However, a mechanism-based study of the interactions between proteins and polyphenols remains important to elucidate the changes in the structure and antioxidant activity in proteins. 

The aim of this study was to develop a new emulsifier with antioxidant capacity. A Pv–GA complex was prepared via a free-radical method and characterized by SDS-PAGE, MALDI-TOF-MS, FTIR, and fluorescence spectroscopy. The antioxidant activity and emulsifying properties of Pv–GA complexes were investigated. Physicochemical information about the formed Pv–GA complex was obtained, including thermodynamic parameters, site numbers, and the affinity constant [20]. Study of the conjugation mechanisms between Pv and GA could provide important information for further research and offer a theoretical basis for the application of the Pv–GA complex. 

## 2. Materials and Methods 

### 2.1. Materials

Chicken eggs were obtained at a local market (Harbin, China). Gallic acid and ABTS were purchased from Solarbio (Beijing, China). The DPPH reagent was obtained from Sigma (St. Louis, MO, USA). Other reagents were obtained from Aladdin (Shanghai, China).

### 2.2. Apparatus

The protein was characterized by a sodium dodecyl sulfate-polyacrylamide gel electrophoresis (SDS-PAGE) gel preparation kit (Solarbio, Beijing, China). A PHS-3C pH meter (Shanghai Instrument Electric Scientific Instrument Co., Ltd., Shanghai, China) was used to measure the pH of the solution. Centrifugation was performed on a CT14D desktop high speed centrifuge (Shanghai Techcomp Scientific Instrument Co., Ltd., Shanghai, China) and a SC-3610 low speed centrifuge (Hefei Zhongke Zhongjia Scientific Instrument Co., Ltd., Hefei, China). An AL-04 electronic analytical balance (Mettler Toledo Instruments Co., Ltd., Shanghai, China) was used to weigh the sample. The sample was dialyzed with an 8000 Da MWCO filter (Spectrum Labs, Los Angeles, CA, USA). The lyophilizer (LyoQuest-85 Plus, Telstar, Terrassa, Spain) was used to lyophilize the sample.

### 2.3. Extraction of Pv

Pv from egg yolk was extracted from egg yolk according to Jiang [21] with some modifications. Briefly, the egg yolk was diluted with an equal amount of ultrapure water. After being stirred in ice water for 1 h, the diluted egg yolk solution was centrifuged at 10,000 *g* for 45 min to gain a precipitate. Then, the precipitate was mixed with 12% (w/w) (NH_4_)_2_SO_4_ and stirred in an ice water bath for 3 h. After standing overnight at 4 °C, the mixture was heated and stirred in a water bath at 80 °C for 15 min and sonicated for 10 min at 600 w. Dialysis was performed in distilled water for 12 h. Then, the obtained mixture was centrifuged at 10,000 *g* for 25 min. The precipitate was lyophilized to generate a Pv powder.

### 2.4. Preparation of Pv–GA Complexes

Pv–GA complexes were prepared with an ascorbic acid/H_2_O_2_ redox pair acting as an initiator system. A total of 20 mL of 10 mg/mL Pv solution was mixed with 0.4 mL of 5 mol/L H_2_O_2_ containing 0.1 g of ascorbic acid and stirred for 2 h. Then, different amounts of GA were introduced into the mixture to ensure that the final concentrations of GA were 0.5, 1.0, 1.5, 2.0, and 2.5 mg/mL. After being stirred for 24 h, the unreacted GA was removed by dialysis (a dialysis bag with 8000 Da molecular weight cutoffs) at 4 °C for 48 h with distilled water until no free GA existed in the system. The resulting solutions were frozen and lyophilized to obtain Pv–GA complex powder for use. Complexes prepared by Pv and the above-mentioned concentration of GA were named Za, Zb, Zc, Zd, and Ze.

### 2.5. Sodium Dodecyl Sulfate Polyacrylamide Gel Electrophoresis 

SDS-PAGE was performed on a 1 mm thick vertical slab gel (BIO CRAFT model BE-210N, Osaka, Japan). A total of 10 μL of Pv and the Pv–GA complex solution (with a concentration of 2 mg/mL) was loaded onto a 12% separation gel and a 5% stacked gel gel in an electrophoresis system [22]. The separation process was performed at a constant voltage of 80 V for stacking gels and 120 V for stacking gels [23,24]. The gel was stained with 0.05% Coomassie Brilliant Blue R-250 in 0.1 M aluminum mordant to more sensitively detect Pv [25] with high phosphorus content. The gel was then destained with an eluent.

### 2.6. MALDI-TOF-MS

MALDI-TOF-MS was performed as follows: Lyophilized Pv and the Pv–GA complex were dissolved in distilled water to prepare 1 mg/mL sample solutions. The sample solution (1 µL) was spotted to the target. The matrix solution (1 µL; saturated sinapinic acid in 50% acetonitrile with 0.1% TFA) was then covered on top of the sample. After being crystallized under air-drying, the sample was measured with a 4800 Plus MALDI-TOF-TOF TM analyzer (SCIEX, Framingham, MA, USA).

### 2.7. Fourier Transform Infrared (FTIR) Spectroscopy

FTIR spectra of the Pv and Pv–GA complexes were recorded in KBr using a Fourier transform infrared spectrometer (Shimadzu, Kyoto, Japan) [26]. The FT–IR spectrometer was scanned in a range of 4000–400 cm^−1^ with a 4 cm^−1^ resolution at ambient temperature. The experiment was repeated three times. 

The PeakFit version 4.12 software (SeaSolve, San Jose, CA, USA) was used to analyze the protein conformation [27].

### 2.8. Fluorescence Spectroscopy

The Pv or Pv–GA complex was dissolved in 0.01 mmol/L of phosphate buffer (pH 7.0) to obtain a 0.15 mg/mL solution. The fluorescence spectrum with an emission wavelength range of 300 to 500 nm was recorded by a fluorescence spectrometer (PerkinElmer LS55, Fremont, CA, USA). The excitation wavelength was set to 280 nm while the slit width of the monochromator was set to 5 nm.

### 2.9. Antioxidant Activity Assay

#### 2.9.1. DPPH Radical Scavenging Ability

The DPPH radical scavenging activity was measured according to the method reported by Duan [28]. A total of 2.0 mL of the 5 mg/mL Pv–GA complex solution was mixed with 2.0 mL of 95% ethanol containing 0.1 mM DPPH. The mixtures were incubated for 40 min without light exposure, and the absorbance was measured at 517 nm. The DPPH radical’s scavenging activity was calculated using the equation:

(1) $$\mathrm{DPPH}\;\mathrm{radical}\;\mathrm{scavenging}\;\mathrm{activity}\;(\%)=\left(1-\frac{{A-A}_{i}}{A_{0}}\right)\times 100\%$$

where *A* is the absorbance of the DPPH ethanol solution mixed with samples, *A*_0_ is the absorbance of the DPPH ethanol solution mixed with 95% ethanol instead of the samples, and *A_i_* is the absorbance of 95% ethanol instead of the DPPH ethanol solution mixed with the samples.

#### 2.9.2. Reducing Power Assay

The ability to reduce iron (III) was evaluated according to Jiang [29]. One milliliter of the 5 mg/mL Pv–GA complex solution was mixed with 2.5 mL of 0.2 M PBS (pH 6.6) and 2.5 mL of 1% (*w/v*) potassium ferricyanide. The mixture was stored at 50 °C for 30 min. 2.5 mL of 10% (*w/v*) trichloroacetic acid was added to the mixture. Then, centrifugation was performed at 3000 *g* for 10 min. A total of 2.5 mL of the supernatant solution was diluted by an equal volume of distilled water and 0.5 mL of 0.1% (*w/v*) ferric chloride solution. The absorbance was recorded at 700 nm after 10 min [30]. 

#### 2.9.3. ABTS Free Radical Scavenging Ability

The antioxidant activity of the Pv–GA complex was measured [31]. The ABTS^+^ radical solution was stored in dark for 16 h. The working solution containing ABTS^+^ radical was obtained by adjusting to an absorbance of 0.7 ± 0.02 at 734 nm with a sodium phosphate buffer (0.1 mol/L, pH = 7.4). Subsequently, 50 μL of the 5 mg/mL Pv–GA complex solution was mixed with 3 mL of the working solution of the ABTS^+^ radical. After being incubated for 10 min, the mixture was stored in the dark for 5 min at room temperature, and the absorbance of the mixtures was determined at 734 nm. The buffer solution was used as a blank control. The following formula was used to calculate the ABTS free radical’s scavenging rate:

(2) $$\mathrm{ABTS}\;\mathrm{free}\;\mathrm{radical}\;\mathrm{scavenging}\;\mathrm{ability}\;(\%)=\left(1-\frac{A}{A_{0}}\right)\times 100\%$$

where *A* is the absorbance of the samples, and *A*_0_ is the absorbance of samples replaced with deionized water.

### 2.10. Measurement of Emulsifying Properties

The emulsifying activity index (*EAI*) and emulsion stability index (*ESI*) were evaluated by the method of Chen [13]. Peanut oil (.2 mL) was mixed with 18 mL of 10 mg/mL Pv–GA complex solution. The mixture was homogenized at 10,000 rpm for 6 min. The emulsion was obtained in the bottom of the container. Ten microliters of the emulsion were added into 8 mL 0.1% (*w/w*) sodium dodecyl sulfonate (SDS) solution. The absorbance at 500 nm of the mixture was recorded, with 0.1% SDS solution as a blank. *EAI* was calculated according to Formula (3):

(3) $$\mathrm{EAI}=2T\times \frac{A_{0}\times N}{C\times (1-\emptyset )\times 10000}$$

where *A*_0_ is the absorbance at 500 nm, *N* is the dilution factor; *T* is 2.303, *C* is the concentration of samples (10 mg/mL), and *∅* is the volume fraction of peanut oil (10%).

After being incubated for 30 min, the absorbance of the emulsion was recorded. The *ESI* was calculated as follows:

(4) $${\mathrm{ESI}\;(m}^{2}/g)\;=\frac{A_{t}}{A_{0}}\times 100$$

where *A_t_* and *A*_0_ are the absorbances at 30 min and time zero, respectively. 

### 2.11. Mechanism of Pv–GA Conjugation

The change in the intrinsic fluorescence intensity of Pv upon the addition of GA was used to evaluate the interactions between Pv and GA. A total of 10 mg/mL of Pv solution was prepared by dissolving Pv in distilled water, and then different concentrations of GA were added to the Pv solution to obtain solutions with GA concentrations of 0.3 × 10^−5^, 0.6 × 10^−5^, 0.9 × 10^−5^, 1.2 × 10^−5^, and 1.5 × 10^−5^ mol/L. The mixture was placed in a water bath at different temperatures (298, 304, and 310 K) for 15 min. Fluorescence spectra was recorded according to the conditions in Section 2.8. Complexes prepared by Pv and the above-mentioned concentration of GA were named Fa, Fb, Fc, Fd, and Fe.

This type of fluorescence quenching can be described by the Stern–Volmer equation [32]:

(5) $$\frac{F_{0}}{F}{=1+K}_{q}\tau_{0}\left[Q\right]{=1+K}_{\mathrm{sv}}\left[Q\right]$$

where *F*_0_ and *F* are the fluorescence intensities in the absence and presence of the quencher. *K_q_* is the quenching rate constant of the bimolecular (L/mol·s). *τ*_0_ is the average lifetime of the molecule without quencher (the average lifetime of the biomacromolecule is about 10^−8^ s), and [*Q*] is the concentration of the quencher (mol/L). *K_SV_* is the dynamic quenching constant of the Stern–Volmer equation (L/mol).

The binding constant was calculated according to Formula (6) [18]:

(6) $$\log\frac{F_{0}-F}{F}{=\mathrm{logK}}_{a}+\mathrm{nlog}\left[Q\right]$$

where *n* and *K_a_* represent the number of binding sites and the binding constant, respectively.

Hydrophobic forces, van der Waals interactions, hydrogen bonds, and electrostatic interactions were considered as the four kind of forces between a small molecule ligand and a bimolecular molecule ligand [33].The enthalpy change (*ΔH*) could be regarded as a constant within the studied temperature range (298 to 310 K). The free energy change (*ΔG*) can be obtained by the following equation:

(7) ΔG=−RTlnK

(8) ΔG=ΔH−T·ΔS.

Formula (9) was obtained by combining Formula (7) and Formula (8):

(9) $$\mathrm{lnK}=-\frac{\Delta H}{\mathrm{RT}}+\frac{\Delta S}{R}$$

where *R* represents the gas constant (8.314 J/(K·mol)), *T* is the experimental temperature (K), and *K* is the binding constant at the corresponding *T*.

### 2.12. Statistical Analysis

The experiments were repeated three times. Data were recorded as the means ± standard deviation. All data were subjected to an Analysis of Variance (ANOVA) test to evaluate the significant differences (*p* < 0.05) between the means.

## 3. Results and Discussion

### 3.1. Characterization of the Pv–GA Complex

#### 3.1.1. SDS-PAGE

**Figure 1:**
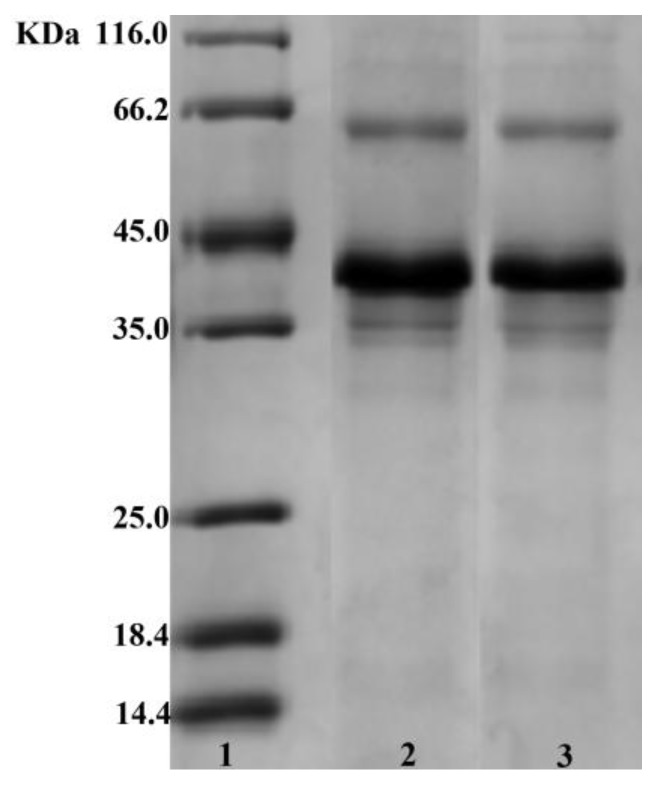
SDS-PAGE analysis of phosvitin (Pv) and the phosvitin-gallic acid (Pv–GA) complex; line 1: marker; Line 2: Pv–GA complex; Line 3: Pv.

The structural changes of Pv after its reaction with GA were monitored by SDS-PAGE (Figure 1). The Pv–GA complex prepared with a GA concentration of 1.5 mg/mL was used to analyze the molecular weight of the Pv–GA complex. The main band in Pv was around 35–45 kDa (line 3). Compared with Pv, there was no obvious change in the molecular mass of the Pv–GA complex (line 2). There are two possible reasons that might explain the constant molecular weight. On the one hand, the molecular weight of GA was too small to cause a change in the molecular weight of Pv. On the other hand, the Pv–GA complex might be destroyed by SDS [34] because SDS can destroy non-covalent bonds, such as hydrogen bonds and hydrophobic bonds.

#### 3.1.2. MALDI-TOF-MS

**Figure 2:**
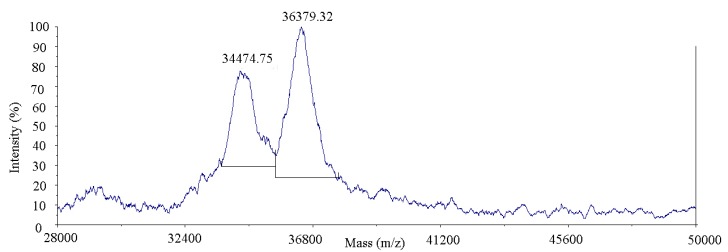
MALDI-TOF-MS analysis of Pv–GA complex.

MALDI-TOF-MS was used to evaluate the change in molecular weight of Pv–GA complex. The molecular mass of the GA was 170.12 Da. The molecular weight of the Pv was 34361.84 Da and 36338.19 Da (As shown in S1 (MALDI-TOF-MS analysis of Pv) in the supplementary materials), which is consistent with literature [35]. The peak of the Pv–GA complex observed in Figure 2, indicating a higher molecular weight than the Pv monomer. The difference in molecular weight between the Pv and the Pv–GA complex might be due to a corresponding insertion of GA molecules into the Pv monomer. The results of MALDI-TOF-MS further confirm the formation of Pv with a GA complex.

#### 3.1.3. FTIR

**Figure 3:**
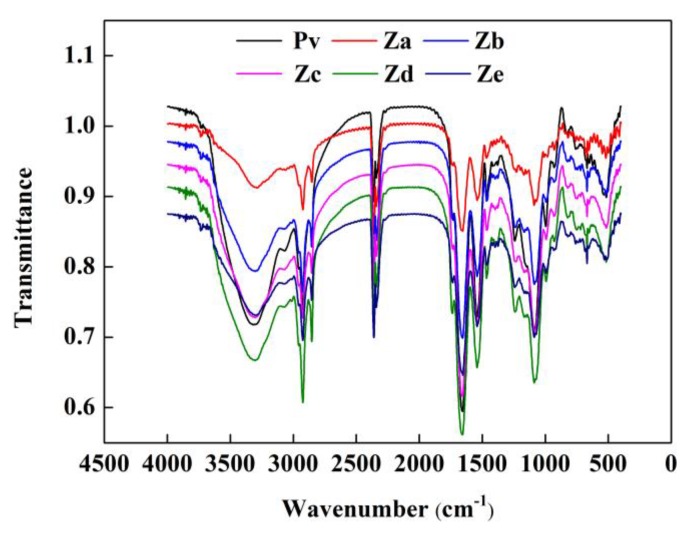
FTIR of Pv–GA complexes.

The Pv–GA complex was characterised by infrared spectroscopy and its derivative methods. The spectral differences between different substances were effectively distinguished by FTIR [36,37]. The infrared spectra of Pv and the Pv–GA complex are shown in Figure 3. The peak at 3301 cm^−1^ represents the stretching vibration of the N–H in the protein. A shift was obtained in the band after the chelation reaction, indicating that the N-H was connected with the formation of the Pv–GA complex. The amide I band at 1660 cm^−11^ in Pv was attributed to the C-N stretching vibration coupled with N–H bending. It shifted to a higher wavenumber in the spectra of the Pv–GA complex. We observed that the intensity was increased for the Pv amide I at 1660 and amide II at 1530 cm^−1^ in the difference spectra of Pv–GA. This result might be due to GA binding to Pv C=O, C–N, and N–H groups (hydrophilic interactions) [19]. These results indicate that C=O, C–N, and N–H groups were also likely to be involved in the formation of the complex [38].

The secondary protein structure of the Pv and Pv–GA complexes was quantitatively analyzed, and the results are shown in Table 1 and Figure S2. It can be seen from Table 1 that with an increase in GA concentration, the content of the α-helix and unordered helix showed a slight change, while the content of the β-sheet and β-turn showed a significant change. The content of the β-sheet first decreased and then increased, and the content of the β-turn gradually decreased. With the addition of GA, the secondary structure of Pv was changed. This indicates a successful conjugation of Pv and GA by a free radical method.

#### 3.1.4. Fluorescence Spectroscopy Analysis

**Figure 4:**
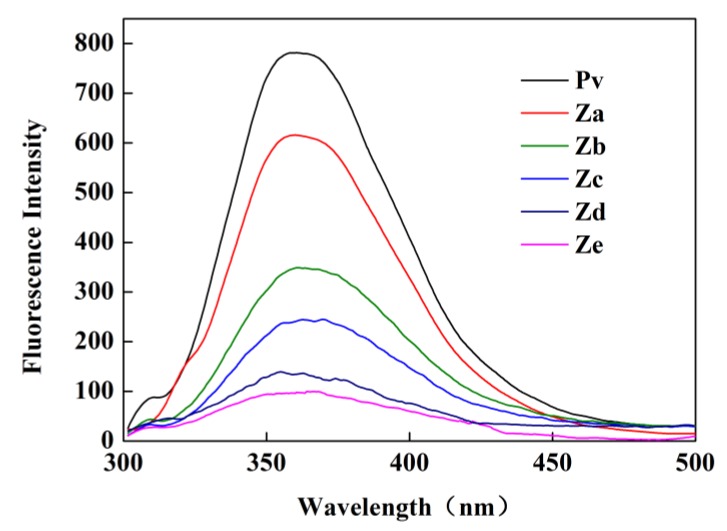
Fluorescence spectroscopy of the Pv–GA complexes.

As indicated in Figure 4, as the concentration of GA increased, a stronger fluorescence quenching effect of the Pv–GA complex was obtained, and there was an offset in the maximum emission wavelength. The maximum emission wavelength of Pv was 358.5 nm. With the addition of GA, the wavelengths were red-shifted to 360, 362.5, 361, 366.5, and 359 nm, respectively. This indicates that the covalent combination of Pv and GA formed a free radical-forming complex via peroxide-ascorbic acid, which might be due to the covalent attachment of tryptophan residues in Pv to the GA. Generally, the redshift was attributed to a greater exposure of the tryptophan residue to the solvent, resulting in a change in the hydrophobic environment of the tryptophan residue [39].

#### 3.1.5. Analysis of Antioxidant Activities

**Figure 5:**
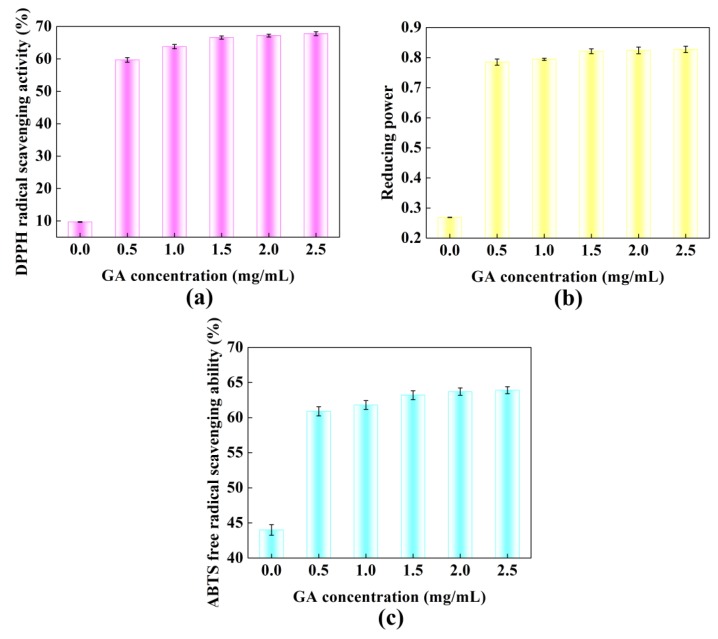
Antioxidant abilities of Pv–GA complexes. (**a**) DPPH radical scavenging; (**b**) reducing power ability; (**c**) ABTS^+^ radical scavenging.

The DPPH test could be used to study the scavenging capability of extracts or different food matrices [40,41]. Figure 5a shows the DPPH scavenging activity changes corresponding to the Pv–GA complexes. When no GA was added, the Pv of the DPPH radical scavenging activity was about 10%. With an increase in GA concentration, the DPPH free radical scavenging ability of the complex increased continuously. Reducing power reflects the capacity to afford electrons or hydrogen. Figure 5b indicates that the reduced power of the Pv–GA complex increased rapidly when the concentrations of GA ranged from 0 to 1.5 mg/mL. The ABTS assay was used to assess the total antioxidant activity. As shown in Figure 5c, the Pv–GA complex showed a better ability for the scavenged the ABTS radical cations compared to Pv. 

The radical scavenging ability of DPPH and ABTS and the reducing power of the Pv–GA complex showed the same trend. When the concentration of GA was less than 1.5 mg/mL, the antioxidant activity of the Pv–GA complex increased. When the concentration of GA was more than 1.5 mg/mL, the antioxidant activity of the Pv–GA complex was basically stable. This result indicates that the conjugation of GA and Pv reached saturation when the GA was at a concentration of 1.5 mg/mL. Furthermore, the antioxidant activity of the Pv–GA complex proved to be much higher than that of Pv.

#### 3.1.6. Analysis of Emulsifying Properties

**Figure 6:**
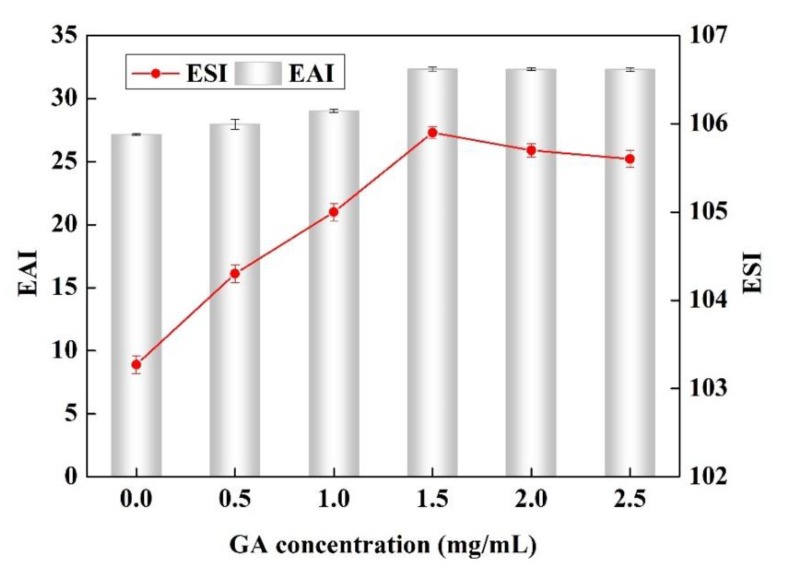
*EAI* and *ESI* of Pv–GA complexes prepared with different concentration of GA.

*EAI* reflects the ability of the emulsifier to rapidly adsorb at the oil/water interface [42]. *ESI* reflects the ability of emulsion droplets to keep dispersed against creaming, coalescing, or flocculation [43]. The *EAI* and *ESI* for the Pv and the Pv–GA complex are shown in Figure 6. When the concentration of GA was 0.0 mg/mL, the Pv showed excellent emulsifying activity, with an *EAI* and *ESI* of 27.14 and 103.27, respectively. As the concentration of GA increased, the trend of *EAI* and *ESI* changed in a similar way. When the concentration of GA was less than 1.5 mg/mL, the Pv–GA complex slightly increased both *EAI* and *ESI*—an effect that was more pronounced as the GA concentration increased. However, *EAI* and *ESI* reached a steady state when the concentration of GA was more than 1.5 mg/mL. These results indicate that the conjugation of Pv with GA slightly improved the emulsifying activity of Pv. The emulsification activity of a protein depends on a number of complex factors, such as the amphiphilic nature and interfacial activity of the protein, as well as the protein’s solubility and mobility [44]. This occurred because of the increased hydrophobicity of Pv after the conjugation of Pv with GA, which could enhance the adsorption ability towards oil [45]. In contrast, Chen [2] indicated that emulsion stabilizing properties was reduced by Pv-Dex, which might be explained by the lower adsorption rate of the protein at the oil/water interface. 

### 3.2. Mechanism of Pv–GA Conjugation

#### 3.2.1. Fluorescence Spectra of Pv Interacting with GA

Tryptophan (Trp), tyrosine (Tyr), and phenylalanine (Phe) residues in proteins can produce fluorescence at a certain excitation wavelength, which is known as the intrinsic fluorescence of proteins. Fluorescence spectroscopy reflects changes in the tertiary structure of proteins [35]. In the current study, Trp and Tyr were simultaneously excited at fluorescence excitation wavelengths of 280 nm. The fluorescence quenching effect of Pv–GA complexes with different GA concentrations was studied by fluorescence spectroscopy. Moreover, binding sites, binding constants, and binding forces for the non-covalent binding of the Pv–GA complex were calculated.

**Figure 7:**
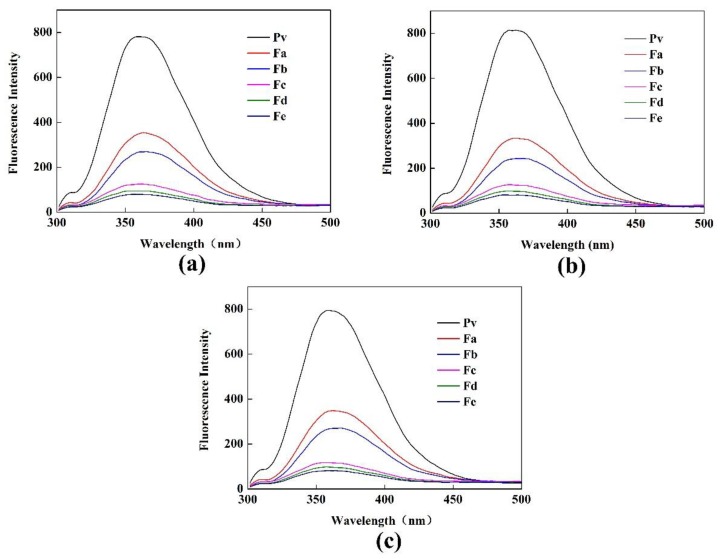
Fluorescence spectroscopy at (**a**) 298 K; (**b**) 304 K; (**c**) 310 K.

The fluorescence spectra of the Pv–GA complexes at 298 K, 304 K, and 310 K are shown in Figure 7. The maximum emission wavelength of Pv was 358 nm, 360.5 nm, and 360 nm at 298 K, 304 K, and 310 K, respectively. Fluorescence intensity decreased with the addition of GA, thereby indicating that the inner fluorescence of Pv was quenched by GA. Moreover, a slight redshift could be observed with an increase of GA content. The fluorescence spectra indicated that the quenching could be caused by the formation of a complex, not only by a dynamic process [46].

#### 3.2.2. Analysis of the Fluorescence Quenching of Pv Induced by GA

Fluorescence quenching generally proceeds according to dynamic quenching or static quenching mechanisms. It can be differentiated by its dependence on temperature [33]. The cause of dynamic annihilation is usually that the collision of the fluorescent substance and the quencher molecule loses energy, or the high concentration of the fluorescent substance causes an increasing collision probability of its own molecule, leading to a loss of energy. Static quenching was mainly caused by the action of fluorescent molecules and quencher molecules to form a complex that did not emit light.

**Figure 8:**
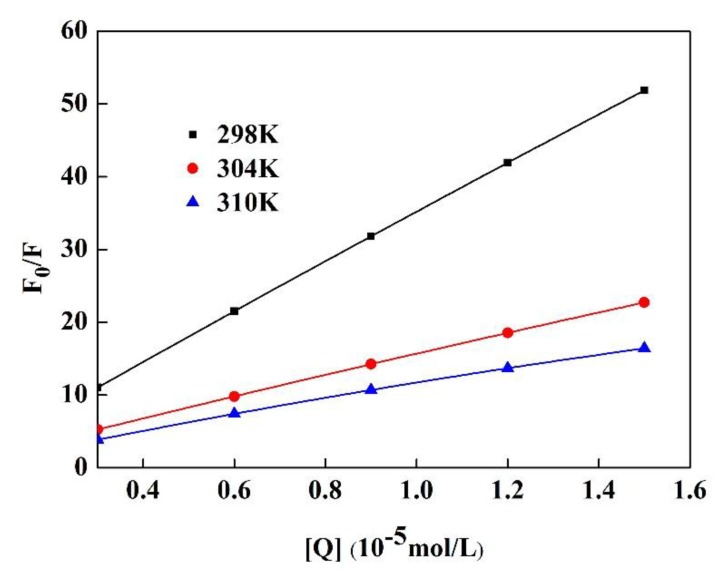
Stern-Volmer plots of Pv quenched by GA at different temperatures.

Figure 8 depicts a Stern-Volmer plot of the fluorescence quenching of Pv induced by GA at 298, 304, and 310 K, while the corresponding *K_sv_* and *K_q_* for the Pv–GA interactions are demonstrated in Table 2.

Table 2 indicates that *K_sv_* gradually decreased with an increasing temperature, meaning that the fluorescence quenching mechanism of Pv by GA was static quenching. Moreover, the experimental *K_q_* was greater than 2 × 10^10^ L/mol·s. The literature [18] suggests that the maximum scatter collision quenching constant of quenchers with the biopolymer is 2 × 10^10^ L/mol·s for dynamic quenching. This further proves that the quenching was not a dynamic annihilation caused by collisions and diffusion between molecules, but a static quenching caused by the conjugation of GA and Pv, which was similar to the quenching that resulted from the formation of the carbamazepine-bovine serum albumin complex [18].

#### 3.2.3. Determination of the Binding Constants and Number of Binding Sites

**Figure 9:**
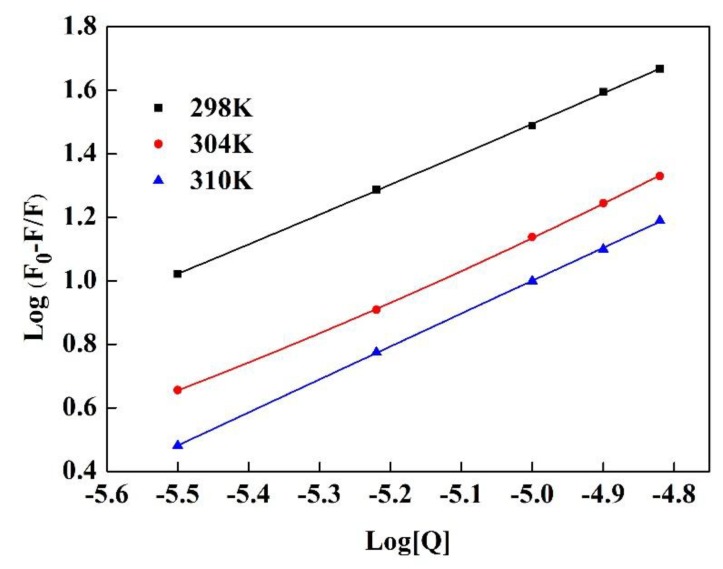
The double-logarithmic curves for the quenching of Pv fluorescence by GA at different temperatures.

The increases of *K_a_* and *n* at different temperatures (Figure 9) are shown Table 3. The results showed that there was a strong interaction between GA and Pv, but the change with the temperature was not obvious. According to the values of *n* that were close to 1, it was inferred that there is an independent class of binding sites on Pv for GA at different temperatures [46]. 

#### 3.2.4. Thermodynamic Parameters and the Nature of the Binding Forces

The thermodynamic parameters were calculated according to Equations (7)–(9). *K* was approximated as the binding constant *K_a_* of the reaction system of the Stern–Volmer equation at the corresponding temperature. The following linear relationship existed between 1/*T* and ln*K*: ln*K* = 6535.4/*T*-7.0167, *R^2^* = 0.9999. The thermodynamic parameters of the Pv–GA interaction are demonstrated in Table 4.

Ross et al. [47] obtained the relationship between the binding properties and the combined thermodynamic function of a small molecule ligand and bimolecular. According to the entropy change (*ΔS*) and *ΔH*, the types of interaction forces of a small molecule ligand and bimolecular ligand were mainly determined. It could be inferred that the interaction process was spontaneous, based on the negative value for *ΔG*, while the negative values of *ΔH* and *ΔS* meant that the main interaction forces were van der Waals interactions and hydrogen bonds [33]. As shown in Table 4, *ΔG* < 0, *ΔH* < 0, and *ΔS* < 0 proved that the reaction was a spontaneous reaction and that the interaction forces of GA and Pv were hydrogen bonds and van der Waals forces.

## 4. Conclusions

In this study, Pv–GA complexes were successfully prepared by a free-radical method, as an emulsifier with an antioxidant capacity. Significant changes in the secondary structure of Pv were obtained after being conjugated with GA. The radical method proved to be an effective approach to prepare a Pv–GA complex with strong antioxidant activity via antioxidant assays. The optimal condition for conjugation was Pv (5 mg/mL) conjugated with 1.5 mg/mL GA. Studies of the mechanisms behind the Pv–GA conjugation indicate that GA could quench the inner fluorescence of Pv. The negative sign for the *ΔG* mean (*ΔG* < 0) shows that the conjugation reaction was a spontaneous reaction. Further, *ΔH* < 0 and *ΔS* < 0 proves that the interaction forces of GA and Pv were hydrogen bonds and van der Waals forces.

## Figures and Tables

**Table 1:** Content of the secondary structure of the Pv–GA complexes.

Samples	*α*-helix	*β*-sheet	*β*-turn	Unordered
Pv	17.11 ± 0.16^a^	27.32 ± 0.14^b^	38.91 ± 0.17^f^	16.66 ± 0.17^a^
Za	18.01 ± 0.14^c^	26.32 ± 0.13^a^	38.31 ± 0.19^e^	17.27 ± 0.17^c^
Zb	18.02 ± 0.12^c^	27.71 ± 0.12^c^	37.31 ± 0.14^d^	16.96 ± 0.17^b^
Zc	18.00 ± 0.12^c^	28.23 ± 0.15^d^	36.90 ± 0.15^c^	16.87 ± 0.17^ab^
Zd	17.92 ± 0.14^ac^	28.71 ± 0.13^e^	36.51 ± 0.18^b^	16.86 ± 0.17^ab^
Ze	17.41 ± 0.15^b^	30.62 ± 0.17^f^	35.20 ± 0.21^a^	16.77 ± 0.17^ab^

**Table 2:** Stern-Volmer constant of the interaction between GA and Pv at different temperatures.

*T/K*	*K_sv_* (L/mol)	*K_q_* (L/mol·s)	*R^2^*
298	3.403 × 10^6^	3.403 × 10^14^	0.9976
304	1.455 × 10^6^	1.455 × 10^14^	0.9953
310	1.045 × 10^6^	1.045 × 10^14^	0.9938

**Table 3:** Binding parameters obtained from the interaction of GA with Pv by a double-logarithmic equation.

*T/K*	*K_a_* (L/mol)	*n*	*R^2^*
298	3.09 × 10^6^	0.9948	0.9978
304	1.53 × 10^6^	0.9956	0.9985
310	1.31 × 10^6^	1.037	0.9999

**Table 4:** The thermodynamic parameters of the Pv–GA interaction.

*T/K*	*ΔH* (kJ/mol)	*ΔG* (kJ/mol)	*ΔS* (J/mol·K)
298	−54.34	−37.02	−58.33
304		−35.99	
310		−35.60	

## Supplementary Materials

The following are available online at https://www.mdpi.com/2073-4360/11/9/1464/s1.

## References

[1] Li X., Li J., Chang C., Wang C., Zhang M., Su Y., Yang Y. (2019). Foaming characterization of fresh egg white proteins as a function of different proportions of egg yolk fractions. Food Hydrocolloid.

[2] Chen H., Jin Y., Ding X., Wu F., Bashari M., Chen F., Cui Z., Xu X. (2014). Improved the emulsion stability of phosvitin from hen egg yolk against different ph by the covalent attachment with dextran. Food Hydrocolloid.

[3] Zhang X., Huang X., Ma M. (2018). Phosphorylated serine clusters of phosvitin plays a crucial role in the regulatory mineralization. Int. J. Biol. Macromol..

[4] Zhang Q., Yang L., Hu S., Liu X., Duan X. (2019). Consequences of ball-milling treatment on the physicochemical, rheological and emulsifying properties of egg phosvitin. Food Hydrocolloid.

[5] Samaraweera H., Zhang W.G., Lee E.J., Ahn D.U. (2011). Egg yolk phosvitin and functional phosphopeptides-review. J. Food Sci..

[6] Duan X., Li M., Ma H., Xu X., Jin Z., Liu X. (2016). Physicochemical properties and antioxidant potential of phosvitin-resveratrol complexes in emulsion system. Food Chem..

[7] Nakamura S., Ogawa M., Nakai S., Kato A., Kitts D.D. (1998). Antioxidant activity of a maillard-type phosvitin-galactomannan conjugate with emulsifying properties and heat stability. J. Agr. Food Chem..

[8] Liu F., Sun C., Yang W., Yuan F., Gao Y. (2015). Structural characterization and functional evaluation of lactoferrin-polyphenol conjugates formed by free-radical graft copolymerization. RSC Adv..

[9] Yuksel Z., Avci E., Erdem Y.K. (2010). Characterization of binding interactions between green tea flavanoids and milk proteins. Food Chem..

[10] Dubeau S., Samson G., Tajmir-Riahi H.-A. (2010). Dual effect of milk on the antioxidant capacity of green, darjeeling, and english breakfast teas. Food Chem..

[11] Badhani B., Sharma N., Kakkar R. (2015). Gallic acid: A versatile antioxidant with promising therapeutic and industrial applications. RSC Adv..

[12] Cao Y., True A.D., Chen J., Xiong Y.L. (2016). Dual role (anti- and pro-oxidant) of gallic acid in mediating myofibrillar protein gelation and gel in vitro digestion. J. Agric. Food Chem..

[13] Chen H., Wu F., Duan X., Yang N., Xu Y., Xu B., Jin Z., Xu X. (2013). Characterization of emulsions prepared by egg yolk phosvitin with pectin, glycerol and trehalose. Food Hydrocolloids.

[14] Liu J., Lu J.F., Kan J., Jin C.H. (2013). Synthesis of chitosan-gallic acid conjugate: Structure characterization and in vitro anti-diabetic potential. Int. J. Biol. Macromol..

[15] Zhang X., Liu J., Qian C., Kan J., Jin C. (2019). Effect of grafting method on the physical property and antioxidant potential of chitosan film functionalized with gallic acid. Food Hydrocolloids.

[16] Gu L., Peng N., Chang C., McClements D.J., Su Y., Yang Y. (2017). Fabrication of surface-active antioxidant food biopolymers: Conjugation of catechin polymers to egg white proteins. Food Biophys..

[17] Curcio M., Puoci F., Iemma F., Parisi O.I., Cirillo G., Spizzirri U.G., Picci N. (2009). Covalent insertion of antioxidant molecules on chitosan by a free radical grafting procedure. J. Agric. Food Chem..

[18] Wang C., Wu Q.H., Wang Z., ZHAO J. (2006). Study of the interaction of carbamazepine with bovine serum albumin by fluorescence quenching method. Anal. Sci..

[19] Kanakis C.D., Hasni I., Bourassa P., Tarantilis P.A., Polissiou M.G., Tajmir-Riahi H.A. (2011). Milk beta-lactoglobulin complexes with tea polyphenols. Food Chem..

[20] Cao X., He Y., Kong Y., Mei X., Huo Y., He Y., Liu J. (2019). Elucidating the interaction mechanism of eriocitrin with β-casein by multi-spectroscopic and molecular simulation methods. Food Hydrocolloids.

[21] Jiang B., Wang L., Wang X., Wu S., Li D., Liu C., Feng Z. (2019). Ultrasonic thermal-assisted extraction of phosvitin from egg yolk and evaluation of its properties. Polymers.

[22] Jiang B., Feng Z.B., Liu C.H., Xu Y.C., Li D.M., Ji G. (2015). Extraction and purification of wheat-esterase using aqueous two-phase systems of ionic liquid and salt. J. Food Sci. Technol..

[23] Jiang B., Yuan Y., Zhang X., Feng Z., Liu C. (2017). Separation and enrichment of lectin from zihua snap-bean (phaseolus vulgaris) seeds by peg 600-ammonium sulfate aqueous two-phase system. Molecules.

[24] Jiang B., Wang L., Na J., Zhang X., Yuan Y., Liu C., Feng Z. Environmentally-friendly strategy for separation of α-lactalbumin from whey by aqueous two phase flotation. Arabian J. Chem..

[25] Zhang X., Qiu N., Geng F., Ma M. (2011). Simply and effectively preparing high-purity phosvitin using polyethylene glycol and anion-exchange chromatography. J. Sep. Sci..

[26] Gao S., Liu Y.Y., Jiang J.Y., Ji Q.Y., Fu Y., Zhao L.X., Li C.Y., Ye F. (2019). Physicochemical properties and fungicidal activity of inclusion complexes of fungicide chlorothalonil with β-cyclodextrin and hydroxypropyl-β-cyclodextrin. J. Mol. Liq..

[27] Jiang B., Na J., Wang L., Li D., Liu C., Feng Z. (2019). Eco-innovation in reusing food by-products: Separation of ovalbumin from salted egg white using aqueous two-phase system of PEG 1000/(NH_4_)_2_SO_4_. Polymers.

[28] Duan X., Zhou Y., Li M., Wu F., Yang N., Xu J., Chen H., Jin Z., Xu X. (2014). Postfertilization changes in conformation of egg yolk phosvitin and biological activities of phosphopeptides. Food Res. Int..

[29] Jiang B., Na J., Wang L., Li D., Liu C., Feng Z. (2019). Separation and enrichment of antioxidant peptides from whey protein isolate hydrolysate by aqueous two-phase extraction and aqueous two-phase flotation. Foods.

[30] Yang H., Deng J., Yuan Y., Fan D., Zhang Y., Zhang R., Han B. (2015). Two novel exopolysaccharides from bacillus amyloliquefaciens c-1: Antioxidation and effect on oxidative stress. Curr. Microbiol..

[31] Zampini I.C., Ordonez R.M., Isla M.I. (2010). Autographic assay for the rapid detection of antioxidant capacity of liquid and semi-solid pharmaceutical formulations using ABTS^+^ immobilized by gel entrapment. AAPS PharmSciTech.

[32] Yang W., Xu C., Liu F., Yuan F., Gao Y. (2014). Native and thermally modified protein-polyphenol coassemblies: Lactoferrin-based nanoparticles and submicrometer particles as protective vehicles for (-)-epigallocatechin-3-gallate. J. Agric. Food Chem..

[33] Wu X., Wu H., Liu M., Liu Z., Xu H., Lai F. (2011). Analysis of binding interaction between (−)-epigallocatechin (EGC) and β-lactoglobulin by multi-spectroscopic method. Spectrochim. Acta A.

[34] Hagerman A.E., Butler L.G. (1980). Condensed tannin purification and characterization of tannin-associated proteins. J. Agric. Food Chem..

[35] Ren J.D., Wu J.P. (2015). Thermal-aided phosvitin extraction from egg yolk. J. Sci. Food Agric..

[36] Wu W., He L., Liang Y., Yue L., Peng W., Jin G., Ma M. (2019). Preparation process optimization of pig bone collagen peptide-calcium chelate using response surface methodology and its structural characterization and stability analysis. Food Chem..

[37] Jiang B., Wang X., Wang L., Lv X., Li D., Liu C., Feng Z. (2019). Two-step isolation, purification, and characterization of lectin from zihua snap bean (phaseolus vulgaris) seeds. Polymers.

[38] Zhang L., Lin Y., Wang S. (2018). Purification of algal calcium-chelating peptide and its physical chemical properties. J. Aquat. Food Prod. T..

[39] Shang L., Wang Y., Jiang J., Dong S. (2007). pH-dependent protein conformational changes in albumin: Gold nanoparticle bioconjugates: A spectroscopic study. Langmuir.

[40] Zhang F., Qu J., Thakur K., Zhang J.G., Mocan A., Wei Z.J. (2019). Purification and identification of an antioxidative peptide from peony (paeonia suffruticosa andr.) seed dreg. Food Chem..

[41] Jiang B., Na J., Wang L., Li D., Liu C., Feng Z. (2019). Reutilization of food waste: One-step extration, purification and characterization of ovalbumin from salted egg white by aqueous two-phase flotation. Foods.

[42] Shevkani K., Singh N., Rana J.C., Kaur A. (2014). Relationship between physicochemical and functional properties of amaranth (amaranthus hypochondriacus) protein isolates. Int. J. Food Sci. Technol..

[43] Feng Z., Li L., Zhang Y., Li X., Liu C., Jiang B., Xu J., Sun Z. (2019). Formation of whey protein isolate nanofibrils by endoproteinase gluc and their emulsifying properties. Food Hydrocolloids.

[44] Lopes-da-Silva J.A., Monteiro S.R. (2019). Gelling and emulsifying properties of soy protein hydrolysates in the presence of a neutral polysaccharide. Food Chem..

[45] Chen L., Chen J., Ren J., Zhao M. (2011). Effects of ultrasound pretreatment on the enzymatic hydrolysis of soy protein isolates and on the emulsifying properties of hydrolysates. J. Agric. Food Chem..

[46] Yu Z., Li D., Ji B., Chen J. (2008). Characterization of the binding of nevadensin to bovine serum albumin by optical spectroscopic technique. J. Mol. Struct..

[47] Ross P.D., Subramanian S. (1981). Thermodynamics of protein association reactions: Forces contributing to stability. J. Am. Chem. Soc..

