# Supplementary material for: Study on the Preparation and Conjugation Mechanism of the Phosvitin-Gallic Acid Complex with an Antioxidant and Emulsifying Capability
Source: Polymers (Basel). 2019 Sep 7;11(9):1464. doi: 10.3390/polym11091464 (PMC6780338; doi:10.3390/polym11091464)
Supplement: Supplementary file 1 [file polymers-11-01464-s001.pdf]

Supplementary Materials

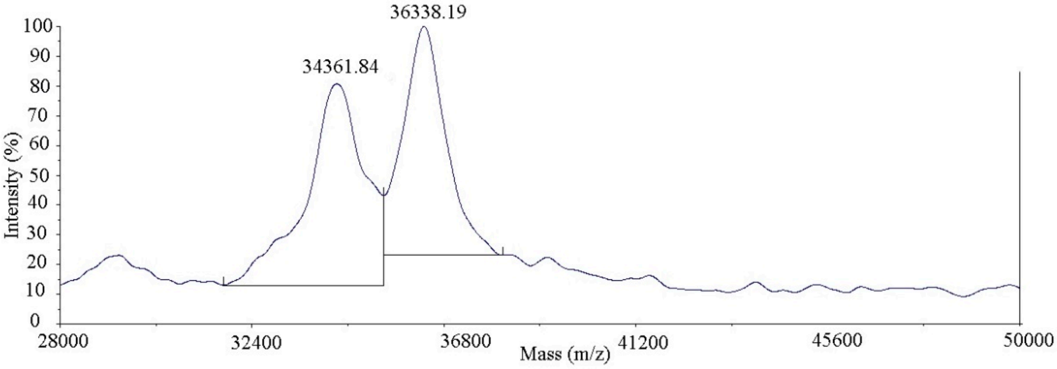

S 1. MALDI-TOF-MS analysis of Pv.

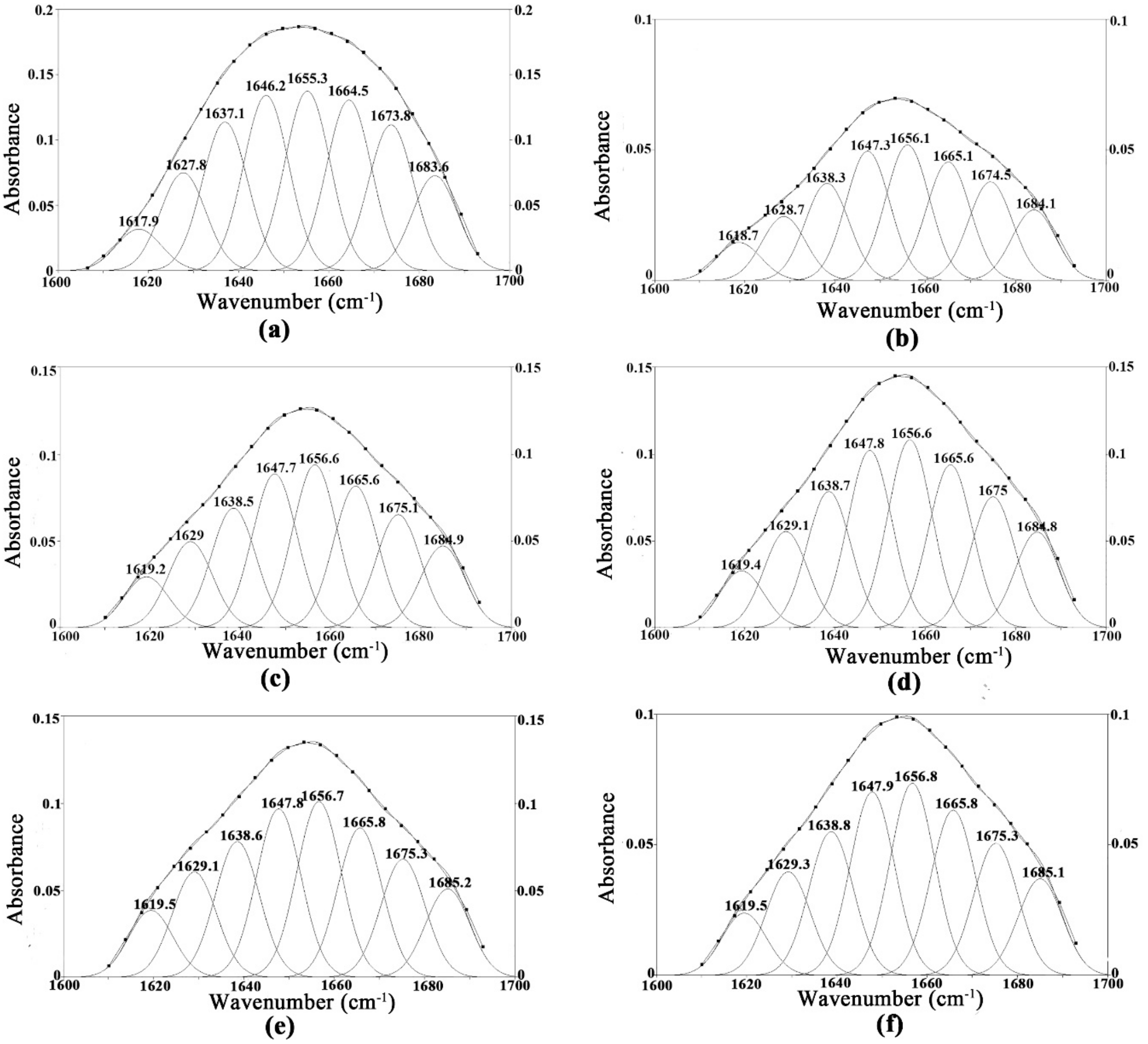

S 2. Second-derivative FTIR spectra in the amide I region and Gaussian curve fitting. (a) Pv; (b)Za; (c)Zb; (d)Zc; (e)Zd; (f)Ze.
